# Species-dependent variation in flavor profile and sensory quality of green tea under aromatic woody intercropping systems

**DOI:** 10.1016/j.fochx.2026.104647

**Published:** 2026-10-16

**Authors:** Yanan Liu, Yanlu He, Liping Yao, Xiangqun Shi, Jiaxin Liu, Li Zhang, Guizhen Chen, Changshuai Yu, Jigang Huang, Yinxiang Gao

**Affiliations:** a College of Pharmacy and Life Science, Jiujiang University, Jiujiang 332005, China; b Jiujiang Agricultural Technology Extension Center, Jiujiang, Jiangxi 332000, China

**Keywords:** Green tea, Aromatic woody intercropping, Companion tree species, Flavor metabolites, Volatile compounds

## Abstract

Aromatic woody intercropping is a promising ecological practice to elevate tea quality, yet how different companion trees differentially modulate tea flavor formation remains unclear. Here, three tea cultivation systems—monoculture (CK), *Osmanthus fragrans* intercropping (EG1), and *Photinia serratifolia* intercropping (EG2)—were constructed to compare green tea flavor traits. We combined sensory evaluation with quantitative detection of amino acids, catechins, caffeine, gallic acid, and semi-quantitative profiling of volatile compounds to profile flavor metabolomes. EG1 enriched umami/sweet amino acids, non-esterified catechins and floral terpene alcohols (linalool, geraniol), associated with fresher taste and more pronounced floral aroma. EG2 accumulated esterified catechins, caffeine, gallic acid and jasmonate-derived volatiles, associated with stronger bitter and astringent sensory characteristics with unique floral notes. We identified 85 volatiles, among which 22 VIP > 1 compounds distinguished cultivation systems. This work reveals distinct associations between companion-tree species and tea flavor-related metabolite profiles under the examined intercropping systems.

## Highlights

• EG1 accumulates umami/sweet amino acids and floral terpenoids to optimize tea quality.
• EG2 boosts bitter and astringent metabolites and generates unique floral scent of tea.
• 22 key volatiles effectively distinguish tea samples from three cultivation planting systems.
• Aromatic woody intercropping reshapes green tea flavor.

## 1 Introduction

Tea (*Camellia sinensis* L.) is one of the most widely consumed non-alcoholic beverages worldwide. Its commercial value is largely determined by flavor quality (Liu et al., 2025; Sun et al., 2024). Amino acids, catechins, caffeine, and volatile compounds collectively shape the sensory characteristics of tea and strongly influence consumer preference and market competitiveness (Cui et al., 2022; Lin et al., 2024; Zan et al., 2025). However, long-term intensive monoculture in tea plantations has led to soil degradation, biodiversity loss, nutrient imbalance, and deterioration of tea quality, thereby posing major challenges to the sustainable development of the tea industry (Wang et al., 2026; Ye et al., 2022). Consequently, environmentally sustainable cultivation strategies simultaneously enhancing ecosystem functioning and tea quality have attracted increasing attention (Gao et al., 2024; Wu et al., 2023).

Tea flavor quality is determined by the coordinated accumulation of volatile and non-volatile metabolites. Volatile compounds impart characteristic floral, fruity, sweet, green, and woody aromas, whereas non-volatile metabolites, including amino acids, catechins, caffeine, and organic acids, collectively determine the taste attributes of tea, such as sweetness, umami, bitterness, and astringency (Wang et al., 2020; Yin et al., 2022; Zhao et al., 2024). Overall sensory quality depends on the dynamic balance among multiple flavor-active metabolites rather than on individual compounds (Zhao et al., 2023). Consequently, cultivation practices that modulate metabolite allocation have become an important approach for improving tea quality.

Tea intercropping has emerged as a promising sustainable cultivation strategy because it can enhance resource use efficiency while simultaneously modifying tea growth and quality. Different agroforestry tree compositions have also been associated with variation in coffee fragrance, aroma, flavor, and overall cup quality (Oliva-Cruz et al., 2024). In cocoa, shade management within agroforestry systems can likewise affect the biochemical and functional characteristics of cocoa beans (Kouassi et al., 2024). Collectively, these findings indicate that the identity, diversity, and management of companion trees can influence the quality-related characteristics of perennial beverage crops.

In tea plantations, intercropping with chestnut, walnut, and various fruit tree species has been demonstrated to alter amino acid accumulation, catechin composition, and volatile profiles, thereby affecting the sensory quality of tea (Wu et al., 2021; Bai et al., 2022; Duan et al., 2024; Wang et al., 2025). However, existing studies have largely focused on individual companion species or specific intercropping systems, whereas comparative evaluations of different aromatic woody species remain limited. In particular, whether and how companion tree identity differentially regulates the accumulation of flavor-related metabolites and volatile compounds in tea remains insufficiently understood.

Previous studies have reported that companion species can influence environmental and biological conditions within tea agroecosystems, including light availability, temperature, humidity, nutrient dynamics, and rhizosphere processes (Wang et al., 2026). These factors have been proposed to affect plant carbon–nitrogen balance and secondary metabolism, which may subsequently influence the accumulation of flavor-related metabolites such as volatile compounds, amino acids, polyphenols, and alkaloids (Wu et al., 2025). Consequently, understanding how different companion species are associated with variations in tea flavor-related metabolites has become an important objective in quality-oriented tea cultivation.

Among potential companion species, aromatic woody plants represent a distinctive group in tea agroforestry systems. Unlike conventional fruit or timber trees, they are characterized by intensive secondary metabolism, persistent emission of aromatic volatiles, complex canopy structures, and diverse ecological interactions with surrounding biotic communities (Zhang et al., 2021; Zhao et al., 2025). These characteristics suggest that aromatic woody species may be associated with distinct metabolic responses in tea plants. However, the ecological and physiological processes underlying such associations remain poorly understood, particularly under field conditions.

In this study, three cultivation systems were compared in the tea plantation, comprising monoculture tea (CK), *O. fragrans* intercropping (EG1), and *P. serratifolia* intercropping (EG2). By integrating metabolomic, volatile compound, and sensory analyses, we sought to determine whether different companion species were associated with distinct changes in tea quality and to identify the metabolic characteristics associated with these intercropping systems.

## 2 Materials and methods

### 2.1 Plant materials and growth conditions

Fresh shoots consisting of one bud and one leaf from the cultivar “Population” were harvested from the Lushan Yunwu tea population on April 5, 2024, at Hailu Tea Expo Park, Jiujiang City, Jiangxi Province, China (116°3′38″ E, 29°34′114″ N). The tea fields were established in 2012. The *Osmanthus fragrans* and *Photinia serratifolia* trees in the two intercropping systems were planted in the same year using two-year-old saplings. At the time of sampling in 2024, both tea plants and companion trees had been growing in the experimental fields for approximately 12 years. Uniform cultivation practices were implemented across all three fields, with no fertilizer applied throughout the trial. The tea plantation was established with a row spacing of 1.5 m, a canopy height of 70–90 cm, and a plant height of 1.0–1.2 m. Three cultivation systems were compared in the tea plantation: monoculture tea (CK), *O. fragrans* intercropping (EG1), and *P. serratifolia* intercropping (EG2). In the CK plot, healthy tea plants were randomly selected, and fresh shoots consisting of one bud with one leaf were collected from each plant. In the EG1 plot, the spacing between the *O. fragrans* trees was 6 m, tea plants growing within a 3-m radius of healthy *O. fragrans* trees were randomly selected and sampled using the same protocol. Similarly, in the EG2 plot, the spacing between the *P. serratifolia* trees was 6 m, tea plants located within a 3-m radius of healthy flowering *P. serratifolia* trees were randomly selected. For each cultivation system, approximately 15 kg of fresh shoots was harvested from numerous tea plants distributed over an area of approximately 0.10 ha. All shoots collected within each cultivation system were pooled to form one composite fresh-leaf sample and then processed as a single batch of green tea. The prepared tea samples were stored at −40 °C until subsequent analyses.

### 2.2 Sensory assessment

Sensory evaluation was performed by five professional panelists with national tea assessor certificates. All panelists met health and visual requirements and followed a unified evaluation protocol to minimize the influence of subjective factors on the results. The assessment included appearance, liquor colour, fragrance, flavor, and infused leaf characteristics, following the Chinese national standard GB/T 23776–2018 and the protocol established by Zhu et al. (2021). For each evaluation, 3 g of tea was infused in 150 mL of freshly boiled water for 4 min. To minimize subjective bias, all samples were assigned randomized numerical codes and evaluated under blind conditions. Sensory attributes, including aroma, liquor colour, and taste, were independently scored and recorded by the panel. All procedures involving sensory evaluation were approved by the Ethics Committee of Jiujiang University (Approval No. JJUM20240090).

### 2.3 Determination of amino acid composition

The composition and concentrations of individual amino acids were determined using ultra-performance liquid chromatography (UPLC) according to previously reported methods with minor modifications (Chen et al., 2025; Liu et al., 2025). Briefly, tea samples were extracted with ultrapure water, followed by vortexing, ultrasonication, and centrifugation. The resulting supernatant was filtered through a 0.22 μm membrane prior to UPLC analysis. Chromatographic separation was performed on a C18 column (100 × 2.1 mm, 1.7 μm) maintained at 35 °C. Detailed information on mobile phase constitution and gradient elution program is provided in Supplementary Method 1.

### 2.4 Determination of catechins, theobromine, gallic acid, and caffeine

The concentrations of catechins, including catechin (C), epicatechin (EC), gallocatechin (GC), catechin gallate (CG), epigallocatechin (EGC), epicatechin gallate (ECG), gallocatechin gallate (GCG), and epigallocatechin gallate (EGCG), as well as theobromine (TB), gallic acid (GA), and caffeine (CAF), were determined by high-performance liquid chromatography (HPLC) following reported methods with minor modifications (Gao et al., 2024; Liu et al., 2025). Briefly, 0.20 g of the powdered tea sample was extracted with 5.0 mL of 70% (*v*/v) aqueous methanol at 70 °C. The mixture was vortexed, incubated in a water bath, and ultrasonically extracted. Following centrifugation (10,000 rpm, 5 min), the collected supernatant was filtered through a 0.22 μm membrane for subsequent analysis.

Chromatographic analysis was performed on an Ultimate 3000 HPLC system (Thermo Fisher Scientific, USA) equipped with a diode-array detector and a Hypersil GOLD Phenyl column (250 × 4.6 mm, 5 μm). The column temperature was maintained at 35 °C, and detection was carried out at 278 nm. Detailed chromatographic conditions, including the mobile phase composition, gradient elution program, and calibration procedures, are provided in Supplementary Method 2. Individual analytes were identified by comparison with authentic standards and quantified using external calibration curves.

### 2.5 Extraction and analysis of volatile compounds using HS-SPME-GC–MS

Volatile compounds were extracted and analyzed using headspace solid-phase microextraction coupled with gas chromatography–mass spectrometry (HS-SPME-GC–MS) according to established methods with minor modifications (Gao et al., 2024; Liu et al., 2025). Volatile constituents were extracted by HS-SPME with a 50/30 μm divinylbenzene/carboxen/polydimethylsiloxane (DVB/CAR/PDMS, 1 cm) fiber (Supelco, Bellefonte, PA, USA). The extracted analytes were subsequently separated and identified using a TSQ 8000 gas chromatography–mass spectrometry system (Thermo Fisher Scientific, Waltham, MA, USA) coupled with a TriPlus RSH autosampler and equipped with a TG-5MS capillary column (30 m × 0.25 mm i.d., 0.25 μm film thickness). Detailed information is provided in Supplementary Method 3.

### 2.6 Qualitative and quantitative analysis of volatile compounds

Volatile compounds were initially identified by comparing the acquired GC–MS total ion chromatograms with the mass spectra available in the National Institute of Standards and Technology (NIST20) mass spectral library. Compounds with a reverse match factor (RSI) > 750 were selected as putatively identified compounds. In addition, a series of n-alkane standards (C7–C40) were analyzed by GC–MS under the same chromatographic conditions described in Section 2.5. The retention indices (RIs) of the detected volatile compounds were calculated based on their retention times and subsequently compared with the literature-reported retention indices (RIL) and with the corresponding RI values obtained using the same or equivalent chromatographic columns available in the NIST Chemistry WebBook for further identification.

Furthermore, several volatile compounds, including benzyl alcohol, linalool, nonanal, and geraniol, were further confirmed by analyzing their corresponding authentic standards under identical GC–MS conditions. The relative concentrations of the volatile compounds were semi-quantitatively determined using ethyl decanoate as the internal standard. Because authentic calibration curves were not established for all detected volatile compounds, the concentrations reported for volatile compounds should be considered semi-quantitative estimates based on the response relative to the internal standard ethyl decanoate. These values were used for comparative profiling among the three cultivation systems rather than as absolute concentrations of individual volatile compounds. The calculation was performed according to the following equation:

$$\mathrm{Wi}=\frac{\mathrm{Si}\times M}{S}$$

where (W_i_) represents the content of the volatile compound; (S_i_) represents the peak area of the volatile compound; (M) represents the amount of the internal standard, ethyl decanoate; and (S) represents the peak area of the internal standard, ethyl decanoate.

### 2.7 ROAVs (relative odor activity values) and TAV (taste activity value) calculation

To clarify the contributions of various volatile components to tea aroma in the present study, ROAVs were computed according to sensory threshold parameters reported in previous literature (Ma et al., 2025). Subsequently, ROAVs were computed via the equation provided below:

$${\mathrm{ROAV}}_{i}=\frac{C_{i}}{{\mathrm{OT}}_{i}}\times \frac{T_{\max}}{C_{\max}}\times 100$$

where C_i_ represents the relative concentration of volatile compounds, and OT_i_ corresponds to the odor threshold of the respective volatile compounds in aqueous solution. C_max_ and T_max_ correspond to the concentration and odor threshold of the volatile compound making the greatest contribution to the overall aroma profile, respectively.

To assess the impact of taste components on the overall taste characteristics of tea samples, TAV was utilized as a key evaluation metric. The subsequent equation was applied for the calculation of TAVs:

$$\mathrm{TAV}=\frac{C}{T}$$

### 2.8 Data processing and statistical analysis

For chemical analyses, each cultivation system was represented by one composite tea sample. Three analytical replicates were performed for non-volatile components and five analytical replicates were performed for volatile compounds. Data are presented as mean ± standard deviation (SD). Because the analytical replicates were derived from the same composite sample, statistical comparisons among cultivation systems were interpreted as descriptive comparisons of the sampled cultivation systems rather than as independently replicated field treatment effects. All results are expressed as mean ± standard deviation (SD). Statistical analyses were conducted using SPSS 27.0 (SPSS Inc., Chicago, IL, USA). Significant differences among treatments were evaluated by one-way analysis of variance (ANOVA), followed by Duncan's multiple range test, with statistical significance set at *P* < 0.05. To characterize the overall variation among tea samples and identify the major metabolites contributing to sample discrimination, principal component analysis (PCA) and partial least-squares discriminant analysis (PLS-DA) were performed using SIMCA 14.1 software (Umetrics, Umea, Sweden). The contribution of individual variables to group discrimination was evaluated using variable importance in projection (VIP) values derived from the PLS-DA model. Data visualization was performed using Origin 2021 (OriginLab Corporation, Northampton, MA, USA) and GraphPad Prism 10.0 (GraphPad Software, San Diego, CA, USA). Hierarchical clustering analysis and heatmap visualization were generated using TBtools 2.131 (accessed on 19 September 2025; https://github.com/CJ-Chen/TBtools).

## 3 Results and discussion

### 3.1 Sensory characteristics

The sensory characteristics of teas produced under monoculture (CK), *O. fragrans* intercropping (EG1), and *P. serratifolia* intercropping (EG2) systems were evaluated based on appearance, liquor colour, aroma, taste, and infused leaves. As shown in Supplementary Table S1, EG1 achieved the highest comprehensive sensory score among the three groups. Compared with CK and EG2, EG1 was characterized by a pronounced umami taste and persistent sweetness, accompanied by mild bitterness and a delicate astringency. The EG2 samples displayed a floral aroma but stronger bitterness and astringency relative to EG1.

### 3.2 Quantitative identification and characterization of non-volatile compounds

#### 3.2.1 Amino acid composition and profile characteristics

The taste of green tea infusion is primarily determined by its non-volatile metabolites. Among them, amino acids are major contributors to sweetness, umami, and bitterness. Amino acids in tea samples from the CK, EG1, and EG2 groups were quantified by HPLC. The results are presented in Fig. 1, and the corresponding ion chromatograms are shown in Fig. S1. The total amino acid concentrations in CK, EG1, and EG2 were 24.78 mg/g, 33.49 mg/g, and 29.80 mg/g, respectively (Supplementary Table S2). Analysis of taste-active amino acids further revealed that the three principal umami-contributing amino acids, glutamic acid (Glu), aspartic acid (Asp), and theanine (The), reached their highest concentrations in EG1. Their relative proportions also peaked in EG1, accounting for 12.26%, 6.61%, and 40.88% of the total amino acids, respectively. In contrast, CK exhibited the lowest proportion of theanine among the three groups (Supplementary Table S4). Similarly, the concentrations of the major sweet-tasting amino acids, including serine (Ser), glycine (Gly), alanine (Ala), and proline (Pro), were significantly higher in EG1 than in CK and EG2. In contrast, histidine (His) accumulated to a significantly greater extent in EG2 than in EG1 (Fig. 1). EG1 exhibited the highest total concentrations of both umami-tasting and sweet-tasting amino acids, reaching 21.18 mg/g and 5.00 mg/g, respectively. EG2 contained the highest level of bitter-tasting amino acids (8.57 mg/g) (Supplementary Table S2).

**Fig. 1:**
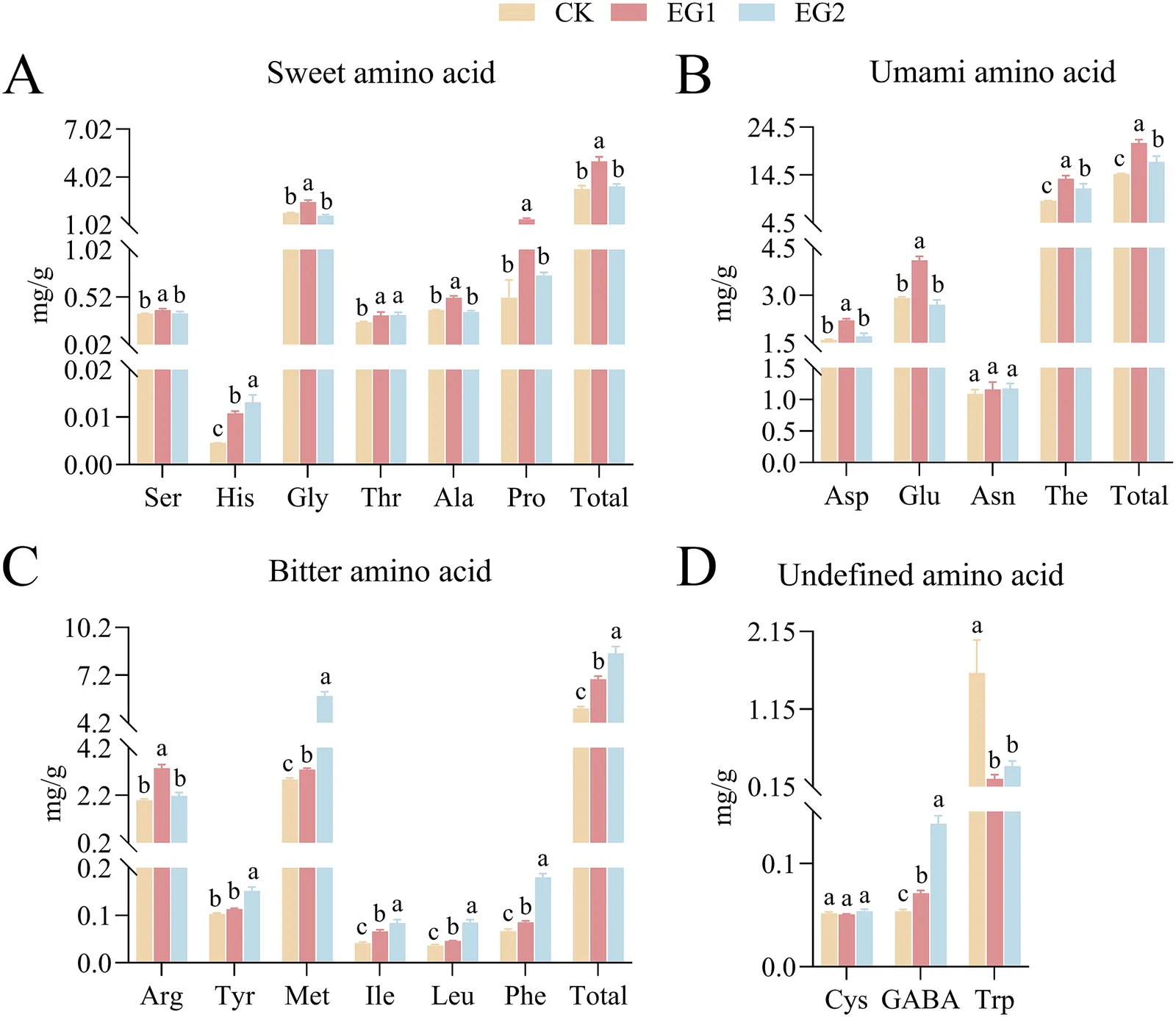
Contents of sweet (A), umami (B), bitter (C) and undefined (D) amino acids (mg/g) detected in CK, EG1 and EG2 green tea by HPLC. Data are presented as means ± standard deviation (*n* = 3). Different lowercase letters indicated significant differences set at *P* < 0.05 based on Duncan's test. Ser, serine; His, histidine; Gly, glycine; Thr, threonine; Ala, alanine; Pro, proline; Asp, aspartate; Glu, glutamate; Asn, asparagine; The, theanine; Arg, arginine; Tyr, tyrosine; Met, methionine; Ile, isoleucine; Leu, leucine; Phe, phenylalanine; Cys, cysteine; Trp, tryptophan; GABA, γ-aminobutyric acid. (For interpretation of the references to colour in this figure legend, the reader is referred to the web version of this article.)

Glutamic acid, aspartic acid, and theanine are well-established contributors to the umami taste of tea. In Longjing green tea, these amino acids, together with histidine, were identified as the principal determinants of umami intensity and exhibited significant positive correlations with sensory umami perception (Deng et al., 2025; Shan et al., 2024). Likewise, alanine, serine, glycine, and proline are recognized as the major sweet-tasting amino acids in tea infusions. Among them, alanine, serine, and glycine accumulate during withering, particularly under moderate temperature and appropriate processing durations, thereby strengthening the sweet and mellow taste of green tea (Ye et al., 2018). Proline remains relatively stable and contributes to the smooth and sweet taste, helping maintain a mellow flavor profile in green tea (Ye et al., 2018). Moreover, the accumulation of alanine and proline under altered light environments has been associated with metabolic reprogramming involved in osmoregulation and stress adaptation (Liu et al., 2025). Although the taste contribution of histidine remains controversial, it is generally classified as a bitter-type amino acid. Some studies suggested that histidine may enhance umami and sweetness rather than impart bitterness at relatively low concentrations, although the underlying mechanisms remain unclear (Shan et al., 2024; Ye et al., 2018). Collectively, the higher abundance of umami- and sweet-tasting amino acids in EG1 provides a chemical basis for its superior sensory quality, characterized by a fresher, sweeter, and more mellow taste.

#### 3.2.2 Catechin, GA, CAF, and TB composition and profile characteristics

The profiles of catechins, caffeine, gallic acid, and theobromine in the CK, EG1, and EG2 tea samples are presented in Fig. 2, and the corresponding ion chromatograms are shown in Fig. S2. Among the esterified catechins (Fig. 2A), all monomers (CG, ECG, GCG, and EGCG) differed significantly among the three treatments. EGCG was the predominant esterified catechin in all samples, followed by ECG, GCG, and CG. Notably, EG2 consistently exhibited the highest concentrations of all esterified catechins, with significantly greater EGCG and GCG concentrations than those observed in CK and EG1. For non-esterified catechins, EGC was the dominant component, followed by EC, C, and GC (Fig. 2B). EG1 showed the highest concentrations of EGC and EC, whereas EG2 exhibited the highest C content but the lowest EGC levels. Total catechin concentrations were significantly higher in EG2 and EG1 than those in CK (Fig. 2C). For esterified catechins, EG2 exhibited the highest level (61.23 mg/g), followed by EG1 (47.60 mg/g) and CK (41.65 mg/g) (Supplementary Table S3). In contrast, EG1 exhibited the highest content of non-esterified catechins (18.06 mg/g), followed by CK (14.10 mg/g) and EG2 (5.49 mg/g) (Supplementary Table S3). These results indicated that EG2 was enriched in esterified catechins, whereas EG1 preferentially accumulated non-esterified catechins. The concentrations of other taste-related metabolites are shown in Fig. 2D. CAF was the most abundant compound, followed by GA and TB. The concentrations of all three metabolites were significantly higher in EG2 than in CK and EG1.

**Fig. 2:**
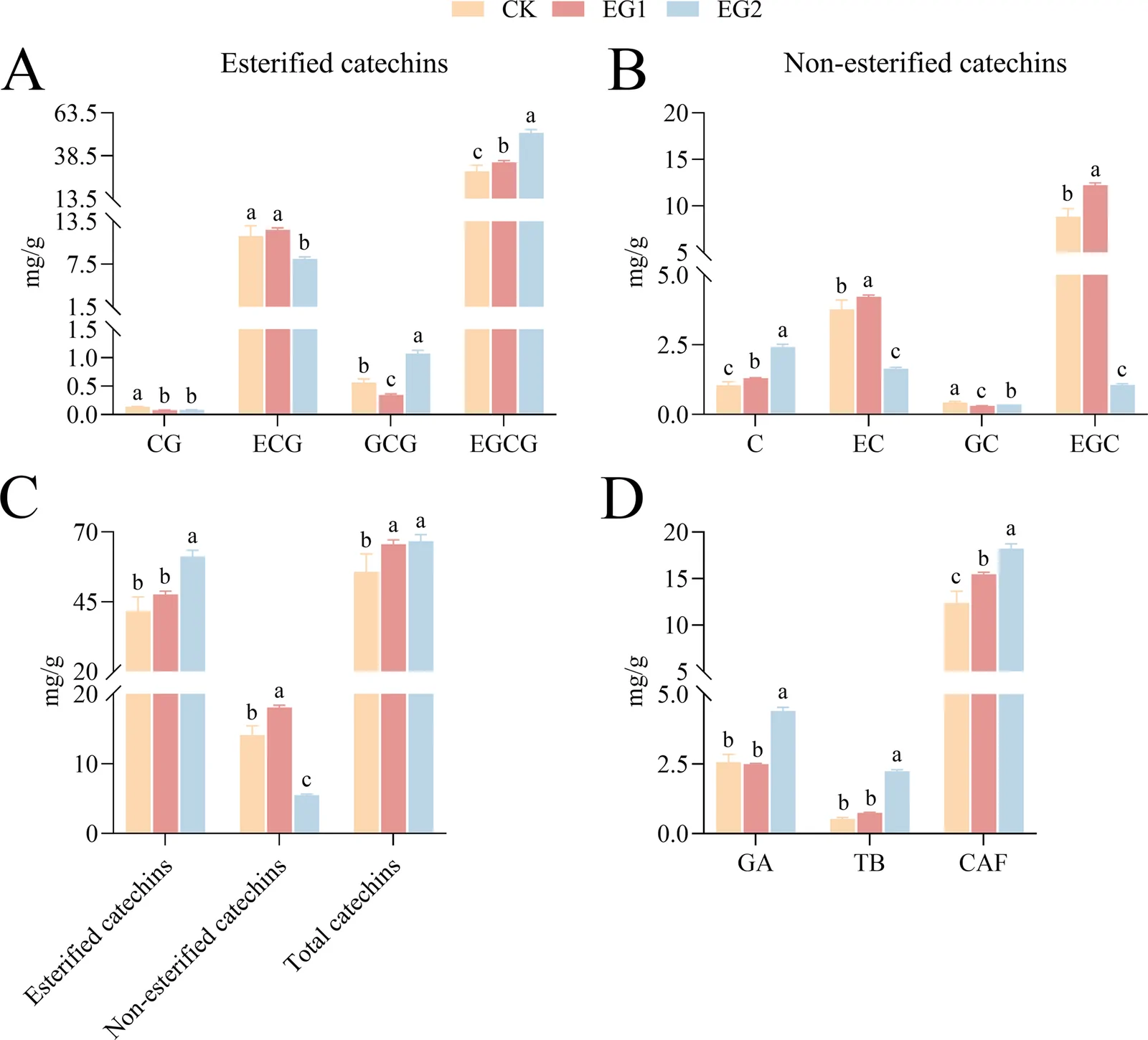
Contents of catechins, gallic acid, caffeine and theobromine (mg/g) detected in CK, EG1 and EG2 green tea by HPLC. (A) Separate esterified catechins (CG, ECG, GCG, EGCG); (B) Separate non-esterified catechins (C, EC, GC, EGC); (C) Total esterified catechins, total non-esterified catechins, and total catechins; (D) Gallic acid (GA), theobromine (TB) and caffeine (CAF). Data are presented as means ± standard deviation (*n* = 3). Different lowercase letters indicated significant differences set at *P <* 0.05 based on Duncan's test. CG, catechin gallate; ECG, epicatechin gallate; GCG, gallocatechin gallate; EGCG, epigallocatechin gallate; C, catechin; EC, epicatechin; GC, gallocatechin; EGC, epigallocatechin; GA, gallic acid; CAF, caffeine; TB, theobromine. (For interpretation of the references to colour in this figure legend, the reader is referred to the web version of this article.)

The distinct metabolite profiles among the three treatments were closely associated with catechin profiles and interspecific interactions. Although both intercropping treatments promoted total catechin accumulation relative to monoculture (CK), they exhibited markedly different catechin composition patterns. EG2 exhibited the highest esterified catechins (61.23 mg/g) and elevated levels of caffeine and gallic acid. Because esterified catechins, particularly EGCG, ECG, and GCG, are the principal contributors to the bitterness and astringency of green tea, their excessive accumulation is likely to diminish the balancing effects of umami amino acids, leading to a heavy and less harmonious flavor profile (Liu et al., 2023; Ye et al., 2022). Collectively, these findings demonstrate that intercropping not only enhanced catechin accumulation but also reshaped catechin composition, favoring non-esterified catechins in EG1 and galloylated catechins in EG2.

Furthermore, the interactions between umami amino acids and catechins play a pivotal role in shaping the overall taste profile of green tea. Umami substances, including theanine, glutamic acid, and aspartic acid, can mitigate the bitterness of esterified catechins through hydrogen bonding, particularly under moderate catechin concentrations (Liu et al., 2023). In addition, non-esterified catechins (EGC and EC) generally impart a milder mouthfeel and reduce excessive astringency (Hou et al., 2026; Liu et al., 2023).

Compared with EG2, EG1 contained lower levels of esterified catechins and higher levels of non-esterified catechins. This compositional pattern may enhance the bitterness-masking effect of umami amino acids while reducing harsh astringency, thereby contributing to a fresher and more balanced taste (Deng et al., 2022; Liu et al., 2023). The preferential accumulation of these amino acids was observed under *O. fragrans* intercropping conditions. Previous studies have suggested that intercropping can influence plant metabolic processes through changes in resource availability and plant–plant interactions, including carbon–nitrogen allocation and nitrogen metabolism (Zan et al., 2025). However, environmental variables such as canopy microclimate, light availability, temperature, humidity, and rhizosphere conditions were not directly measured in the present study. Therefore, their potential contributions to the observed metabolic differences should be regarded as hypotheses rather than demonstrated mechanisms. Within the scope of the present data, the results indicate that *O. fragrans* and *P. serratifolia* intercropping were associated with distinct catechin and amino acid profiles. In particular, *P. serratifolia* intercropping was associated with a higher relative abundance of esterified catechins, whereas *O. fragrans* intercropping was associated with a compositional profile characterized by higher levels of non-esterified catechins and amino acids. These metabolic differences were consistent with the sensory evaluation results, with *O. fragrans* intercropping showing superior sensory quality compared with *P. serratifolia* intercropping. Further studies incorporating direct measurements of canopy microclimate, soil/rhizosphere conditions, and plant physiological responses are required to determine whether these ecological factors contribute causally to the observed metabolic shifts.

### 3.3 TAV analysis of key taste-active compounds

To further evaluate the contributions of individual metabolites to the sensory characteristics of tea infusions, the TAVs of 30 taste-related compounds were calculated (Table 1). Among these compounds, 19 exhibited TAVs greater than 1, including C, EC, GC, EGC, CG, ECG, GCG, EGCG, GA, CAF, TB, Asp, Glu, Asn, Arg, Pro, The, Cys and Met. Among the bitter-related compounds, EG2 exhibited the highest TAVs for the major catechins, particularly EGCG, GCG, and C. EGCG exhibited the highest TAV (595.93). Likewise, the TAVs of GA, CAF, and TB were significantly higher in EG2 than in EG1 and CK. As the principal bitter alkaloid, CAF exhibited TAVs in the order of EG2 (187.872) > EG1 (159.416) > CK (127.642). CAF and EGCG formed the major chemical basis for the pronounced bitter taste in EG2.

**Table 1:** TAV analysis of taste components in CK, EG1 and EG2 green tea samples.

Compound	Taste characteristic	Threshold mg/g		TAV	
			CK	EG1	EG2
C	Bitter, Astringent	0.119	8.86 ± 1c	10.96 ± 0.13b	20.36 ± 0.78a
EC	Astringent	0.27	13.95 ± 1.25b	15.63 ± 0.24a	6.08 ± 0.17c
GC	Astringent	0.165	2.6 ± 0.25a	1.9 ± 0.05c	2.23 ± 0b
EGC	Astringent	0.159	55.66 ± 5.39b	76.9 ± 1.47a	6.67 ± 0.25c
CG	Bitter, Astringent	0.111	1.24 ± 0.07a	0.71 ± 0.02b	0.75 ± 0.04b
ECG	Bitter, Astringent	0.115	99.3 ± 12.71a	107.23 ± 2.26a	71.58 ± 2.48b
GCG	Astringent	0.179	3.14 ± 0.37b	1.95 ± 0.1c	5.99 ± 0.32a
EGCG	Bitter, Astringent	0.087	339.47 ± 40.18c	400.46 ± 10.23b	595.93 ± 22.06a
GA	Bitter, Astringent	0.034	75.41 ± 8.07b	73.15 ± 0.94b	129.4 ± 3.85a
CAF	Bitter	0.097	127.64 ± 12.8c	159.42 ± 2.36b	187.87 ± 5.46a
TB	Bitter	0.114	4.6 ± 0.47c	6.51 ± 0.14b	19.64 ± 0.46a
Asp	Umami	1	1.59 ± 0.02b	2.21 ± 0.04a	1.7 ± 0.1b
Glu	Umami	0.3	9.71 ± 0.13b	13.69 ± 0.42a	9.01 ± 0.5b
Asn	Umami	1	1.09 ± 0.07a	1.16 ± 0.11a	1.17 ± 0.08a
Ser	Sweet	1.5	0.23 ± 0b	0.26 ± 0.01a	0.23 ± 0.01b
His	Sweet	0.2	0.02 ± 0c	0.05 ± 0b	0.07 ± 0.01a
Gly	Sweet	2.6	0.67 ± 0.01b	0.93 ± 0.05a	0.61 ± 0.03b
Arg	Bitter	0.5	3.98 ± 0.13b	6.69 ± 0.31a	4.37 ± 0.27b
Thr	Sweet	0.6	0.43 ± 0.01b	0.54 ± 0.06a	0.55 ± 0.05a
Ala	Sweet	3	0.13 ± 0b	0.17 ± 0.01a	0.12 ± 0.01b
Pro	Sweet	1.3	0.4 ± 0.14b	1.03 ± 0.06a	0.58 ± 0.02b
The	Umami	0.06	151.92 ± 0.35c	228.27 ± 11.4a	194.37 ± 16.5b
Cys	Astringent	0.02	2.59 ± 0.07a	2.54 ± 0.03a	2.68 ± 0.1a
Tyr	Bitter	0.906	0.11 ± 0b	0.12 ± 0b	0.17 ± 0.01a
GABA	Mouth-Drying, Astringent	2	0.03 ± 0c	0.04 ± 0b	0.07 ± 0a
Met	Bitter	0.3	9.54 ± 0.24c	10.95 ± 0.21b	19.64 ± 0.89a
Ile	Bitter	0.9	0.05 ± 0c	0.07 ± 0b	0.09 ± 0.01a
Leu	Bitter	1.9	0.02 ± 0c	0.02 ± 0b	0.04 ± 0a
Phe	Bitter	0.9	0.07 ± 0.01c	0.09 ± 0b	0.2 ± 0.01a
Trp	Bitter	–	–	–	–

Among the three treatments, EG1 contained the greatest number of amino acids with TAVs >1, with eight compounds exceeding the taste threshold, compared with seven compounds in both CK and EG2. Meanwhile, EG1 was the only treatment that encompassed all four major taste attributes—umami, sweetness, bitterness, and astringency—whereas CK and EG2 exhibited only three taste attributes (umami, bitterness, and astringency), with no detectable contribution from sweet-tasting amino acids. The TAVs of the major umami-related amino acids, including Asp, Glu, and The, were consistently highest in EG1. Among them, theanine exhibited the highest TAV (228.27), highlighting its dominant contribution to the umami taste of EG1. Sweetness can enhance the mellow and refreshing characteristics of tea while mitigating bitterness and astringency. Proline (Pro), a representative sweet-tasting amino acid, exhibited a TAV greater than 1 (1.03) only in EG1, whereas its TAV remained below the taste threshold in CK and EG2. In contrast, methionine, an amino acid associated with astringency, was the highest in EG2. Consequently, EG2 was characterized by stronger bitterness and astringency, whereas EG1 exhibited reduced bitterness and astringency with enhanced umami and freshness, leading to a fresher and mellower taste.

### 3.4 Quantitative identification and characterization of volatile compounds

Aroma is a key determinant of green tea quality and is largely governed by the composition and abundance of volatile compounds. HS-SPME-GC–MS identified a total of 85 volatile components in CK, EG1, and EG2 samples (Table 2). These compounds were subsequently subjected to hierarchical cluster analysis (HCA). The corresponding heatmap is presented in Fig. 3. The HCA clearly discriminated between the three treatments. CK formed an independent cluster, while EG1 and EG2 were grouped together. Notably, hydrocarbons were the predominant volatile class in CK samples.

**Table 2:** Information on volatile compounds identified in CK, EG1, and EG2 green tea samples by GC–MS.

	**RT**	**NAME**	**CAS**	**RIa**	**RIb**	**ID**	**Contents (μg/kg)**		
							**CK**	**EG1**	**EG2**
1	7.57	Styrene	100–42-5	897	893	MS,RI	3.33 ± 0.45a	3.52 ± 0.18a	1.8 ± 0.65b
2	7.82	Heptanal	111–71-7	905	901	MS,RI	–	20.41 ± 2.88a	5.01 ± 0.63b
3	8.72	α-Pinene	80–56-8	936	937	MS,RI	–	19.55 ± 2.27a	–
4	9.04	3-Ethyl-3-methylheptane	17,302–01-1	947	953	MS,RI	2.96 ± 0.68a	3.02 ± 0.3a	2.5 ± 0.73a
5	9.96	β-Pinene	127–91-3	979	979	MS,RI	–	28.8 ± 2.9a	19.57 ± 2.52b
6	10.34	β-Myrcene	123–35-3	992	991	MS,RI,STD	0.99 ± 0.24b	1.86 ± 0.42a	0.74 ± 0.33b
7	11.67	Limonene	138–86-3	1029	1030	MS,RI	5.7 ± 0.97b	6.82 ± 0.3a	4.53 ± 0.85c
8	12.13	Benzyl alcohol	100–51-6	1041	1037	MS,RI,STD	0.31 ± 0.18b	0.64 ± 0.18b	1.63 ± 0.69a
9	12.33	trans-β-Ocimene	3779-61-1	1047	1049	MS,RI	2.24 ± 0.35b	2.95 ± 0.51b	7.66 ± 1.15a
10	12.47	1H-Pyrrole-2-carboxaldehyde, 1-ethyl-	2167-14-8	1051	1046	MS,RI	0.73 ± 0.18b	0.72 ± 0.05b	3.64 ± 1.26a
11	12.63	Decane, 2,6,7-trimethyl-	62,108–25-2	1055	1058	MS,RI	12.18 ± 1.62a	9.8 ± 1.35b	6.5 ± 1.52c
12	12.85	Decane, 2-methyl-	6975-98-0	1061	1064	MS,RI	4.06 ± 0.72a	3.49 ± 0.86a	1.87 ± 0.56b
13	13.2	1-Octanol	111–87-5	1070	1071	MS,RI	6.13 ± 1.33a	4.41 ± 0.77b	6.34 ± 1.29a
14	13.3	cis-Linalool oxide	5989-33-3	1072	1074	MS,RI	16.11 ± 1.96a	14.3 ± 1.32a	14.22 ± 2.03a
15	13.95	trans-Linalool oxide (furanoid)	34,995–77-2	1090	1086	MS,RI	18.67 ± 2.77b	22.71 ± 2.54a	15.29 ± 2.85b
16	14.16	Undecane	1120-21-4	1095	1100	MS,RI	3.46 ± 0.81a	3.52 ± 0.64a	2.19 ± 0.65b
17	14.38	Linalool	78–70-6	1101	1099	MS,RI,STD	89.28 ± 11.08a	101.19 ± 11.3a	40.3 ± 7.42b
18	14.58	Nonanal	124–19-6	1105	1104	MS,RI,STD	35.68 ± 5.21a	37.44 ± 3.21a	40.12 ± 7.82a
19	15.36	Phenylethyl Alcohol	60–12-8	1122	1116	MS,RI,STD	0.8 ± 0.17c	15.44 ± 1.16b	26.43 ± 3.81a
20	15.54	Decane, 3,7-dimethyl-	17,312–54-8	1125	1125	MS,RI	3.9 ± 1.15b	7.98 ± 0.87a	5.37 ± 1.47b
21	17.25	Undecane, 2-methyl-	7045-71-8	1162	1164	MS,RI	2.54 ± 0.49a	2.62 ± 0.67a	2.18 ± 0.39a
22	17.62	Undecane, 3-methyl-	1002-43-3	1169	1170	MS,RI	5.8 ± 0.76b	6.88 ± 1.07ab	7.3 ± 0.48a
23	17.85	trans-Linalool oxide (pyranoid)	39,028–58-5	1174	1173	MS,RI	37.09 ± 5.86b	31.07 ± 3.44b	46.25 ± 6.54a
24	18.43	Butanoic acid, 3-hexenyl ester, (*Z*)-	16,491–36-4	1186	1187	MS,RI	4 ± 0.51b	1.02 ± 0.37c	6.52 ± 0.78a
25	18.72	α-Terpineol	98–55-5	1193	1189	MS,RI,STD	16.47 ± 2.2a	7.94 ± 0.63b	5.06 ± 0.81c
26	19.05	Dodecane	112–40-3	1200	1200	MS,RI	33.7 ± 3.91b	48.67 ± 2.97a	47.93 ± 4.91a
27	19.74	Undecane, 3,6-dimethyl-	17,301–28-9	1212	1210	MS,RI	2.74 ± 0.65a	1.76 ± 0.41b	1.51 ± 0.32b
28	20.27	β-Cyclocitral	432–25-7	1222	1220	MS,RI	–	2.63 ± 0.63a	2.45 ± 0.46a
29	22.03	Geraniol	106–24-1	1255	1255	MS,RI,STD	37.23 ± 5.95b	67.13 ± 6.78a	22.63 ± 3.47c
30	22.45	Dodecane, 2-methyl-	1560-97-0	1262	1264	MS,RI	3.31 ± 0.36a	2.25 ± 0.67b	2.63 ± 0.38b
31	22.97	Citral	5392-40-5	1272	1276	MS,RI	–	3.33 ± 0.2a	–
32	23.34	Dodecane, 2,6,11-trimethyl-	31,295–56-4	1279	1275	MS,RI	29.63 ± 2.33a	20.06 ± 2.37b	17.97 ± 1.61b
33	24.12	Tridecane	629–50-5	1293	1300	MS,RI	5.88 ± 0.88a	3.31 ± 0.56b	3.15 ± 0.52b
34	24.47	Indole	120–72-9	1300	1295	MS,RI	12.95 ± 3.28b	15.4 ± 0.89b	38.9 ± 5.93a
35	24.8	1-Octanol, 2-butyl-	3913-02-8	1306	1277	MS,RI	15.98 ± 1.61a	9.69 ± 1.07b	8.15 ± 1b
36	25.93	Dodecane, 4,6-dimethyl-	61,141–72-8	1325	1325	MS,RI	36.08 ± 3.49a	24.61 ± 2.82b	22.36 ± 2.41b
37	26.68	σ-Elemene	20,307–84-0	1338	1338	MS,RI	–	–	0.85 ± 0.18a
38	27.02	Dodecane, 2,6,10-trimethyl-	3891-98-3	1344	1366	MS,RI	18.94 ± 3.41a	9.01 ± 1.39b	6.75 ± 0.73b
39	27.36	α-Cubebene	17,699–14-8	1350	1351	MS,RI	–	5.2 ± 0.85b	8.19 ± 1.01a
40	27.49	Naphthalene, 1,2-dihydro-1,1,6-trimethyl-	30,364–38-6	1352	1354	MS,RI	8.42 ± 0.92a	6.13 ± 0.85b	7.6 ± 0.98a
41	28.08	Tridecane, 2-methyl-	1560-96-9	1363	1364	MS,RI	2.01 ± 0.32a	1.67 ± 0.52ab	1.46 ± 0.22b
42	28.47	Tridecane, 3-methyl-	6418-41-3	1369	1371	MS,RI	2.42 ± 0.61a	1.99 ± 0.55a	1.85 ± 0.14a
43	28.87	Copaene	3856-25-5	1376	1376	MS,RI	2.51 ± 0.29b	4.16 ± 0.54a	2.8 ± 0.37b
44	29.17	cis-3-Hexenyl hexanoate	31,501–11-8	1382	1380	MS,RI,STD	79.36 ± 11.62a	43.77 ± 3.87b	74.01 ± 9.51a
45	29.8	trans-2-Hexenyl caproate	53,398–86-0	1393	1391	MS,RI	5.21 ± 1.39c	9.01 ± 0.84b	13.41 ± 1.12a
46	30.21	cis-Jasmone	488–10-8	1400	1394	MS,RI,STD	37.46 ± 4.08b	32.34 ± 1.94b	88.08 ± 9.83a
47	31.37	β-Caryophyllene	87–44-5	1420	1419	MS,RI,STD	–	16.51 ± 1.47a	6.44 ± 0.88b
48	31.93	β-Copaene	18,252–44-3	1430	1432	MS,RI	–	1.68 ± 0.44a	–
49	32.21	γ-Elemene	29,873–99-2	1434	1433	MS,RI	–	–	5.9 ± 0.39a
50	32.93	Cyclopentane, nonyl-	2882-98-6	1447	1451	MS,RI	3.92 ± 0.32a	4.86 ± 1.57a	4.31 ± 0.82a
51	33.32	Geranyl acetone	689–67-8	1454	1456	MS,RI,STD	1.87 ± 0.3b	4.49 ± 1a	2.19 ± 0.38b
52	33.54	(*E*)-β-Famesene	18,794–84-8	1457	1457	MS,RI	8.53 ± 1.25a	6.73 ± 0.35b	6.46 ± 0.37b
53	33.77	2,6,10-Trimethyltridecane	3891-99-4	1461	1449	MS,RI	22.69 ± 2.64a	19.35 ± 1.46b	14.48 ± 0.8c
54	33.88	cis-Muurola-4(15),5-diene	157,477–72-0	1463	1463	MS,RI	–	6.45 ± 0.49a	–
55	34.08	2,6-Di-tert-butylbenzoquinone	719–22-2	1467	1471	MS,RI	1.21 ± 0.48a	0.46 ± 0.21b	0.59 ± 0.33b
56	34.26	Tetradecane, 3-methyl-	18,435–22-8	1470	1470	MS,RI	4.04 ± 0.43a	2.76 ± 0.67b	2.1 ± 0.13c
57	34.69	γ-Muurolene	30,021–74-0	1477	1477	MS,RI	0.86 ± 0.41c	5.72 ± 1.03a	2.26 ± 0.37b
58	34.9	Germacrene D	23,986–74-5	1481	1481	MS,RI	–	2.8 ± 0.69a	3.03 ± 0.3a
59	35.24	trans-β-Ionone	79–77-6	1487	1486	MS,RI	9.82 ± 1.24a	10.12 ± 1.2a	5.45 ± 0.46b
60	35.53	Bicyclo[4.4.0]dec-1-ene, 2-isopropyl-5-methyl-9-methylene-	150,320–52-8	1492	1507	MS,RI	–	1.26 ± 0.24b	2.14 ± 0.26a
61	35.97	Pentadecane	629–62-9	1499	1500	MS,RI	48.45 ± 4.84a	53.2 ± 6.88a	31.99 ± 2.48b
62	36.52	α-Farnesene	502–61-4	1509	1508	MS,RI,STD	8.04 ± 1.2a	4.3 ± 0.45b	3.75 ± 0.49b
63	36.83	γ-Cadinene	39,029–41-9	1515	1513	MS,RI	5.49 ± 0.67b	10.05 ± 1.91a	4.93 ± 0.26b
64	37.36	δ-Cadinene	483–76-1	1524	1524	MS,RI	17.55 ± 3.17b	38.23 ± 7.18a	34.44 ± 3.65a
65	37.73	Dihydroactinidiolide	17,092–92-1	1530	1532	MS,RI	3.46 ± 1.51a	3.37 ± 1.07a	0.19 ± 0.04b
66	38.28	Tetradecane, 2,6,10-trimethyl-	14,905–56-7	1540	1539	MS,RI	23.04 ± 1.94a	13.32 ± 1.58b	13.09 ± 1.2b
67	38.47	α-Calacorene	21,391–99-1	1544	1542	MS,RI	0.51 ± 0.25b	1.7 ± 0.34a	1.69 ± 0.14a
68	38.84	1-Dodecanol, 3,7,11-trimethyl-	6750-34-1	1550	1571	MS,RI	11.38 ± 1.16a	7.75 ± 0.41b	8.03 ± 0.77b
69	39.63	*E*-Nerolidol	40,716–66-3	1564	1564	MS,RI,STD	69.27 ± 8.47a	49.89 ± 5.21b	25.57 ± 0.94c
70	39.97	Dodecane, 5,8-diethyl-	24,251–86-3	1570	1572	MS,RI	9.09 ± 0.96a	6.23 ± 1.16b	4.77 ± 0.63c
71	40.11	E-2-Hexenyl benzoate	76,841–70-8	1573	1588	MS,RI	6.33 ± 1.18a	2.28 ± 0.16c	3.46 ± 0.73b
72	41.48	Diethyl Phthalate	84–66-2	1597	1594	MS,RI	2.09 ± 1.25a	1.61 ± 0.29ab	0.82 ± 0.53b
73	41.63	Hexadecane	544–76-3	1599	1600	MS,RI	40.62 ± 6.05a	33.98 ± 4.02b	29.6 ± 1.85b
74	42.95	α-Corocalene	20,129–39-9	1623	1623	MS,RI	–	0.99 ± 0.13a	0.88 ± 0.05a
75	43.25	cubenol	21,284–22-0	1629	1642	MS,RI	–	–	2.63 ± 0.22a
76	43.99	τ-Cadinol	5937-11-1	1642	1640	MS,RI	4 ± 0.88b	3.35 ± 0.83b	5.56 ± 0.6a
77	44.39	Methyl jasmonate	1211-29-6	1650	1638	MS,RI	3.66 ± 1.02b	3.28 ± 1.07b	15.49 ± 0.79a
78	44.58	Cyclopentane, undecyl-	6785-23-5	1653	1660	MS,RI	2.5 ± 0.3b	4.77 ± 0.75a	1.51 ± 0.42c
79	44.68	α-Cadinol	481–34-5	1655	1653	MS,RI	1.19 ± 0.22b	1.87 ± 0.71a	1.96 ± 0.23a
80	45.79	Naphthalene, 1,6-dimethyl-4-(1-methylethyl)-	483–78-3	1675	1674	MS,RI	0.7 ± 0.35b	1.5 ± 0.28a	0.99 ± 0.13b
81	47.11	Heptadecane	629–78-7	1699	1700	MS,RI	5.79 ± 0.8a	5.04 ± 1.15ab	4.04 ± 0.39b
82	52.35	Hexadecane, 2,6,10,14-tetramethyl-	638–36-8	1799	1792	MS,RI	2.94 ± 0.3a	2.45 ± 0.31b	1.78 ± 0.23c
83	54.33	Neophytadiene	504–96-1	1839	1837	MS,RI	3.21 ± 0.39b	5.31 ± 0.69a	1.03 ± 0.33c
84	58.75	Hexadecanoic acid, methyl ester	112–39-0	1928	1926	MS,RI,STD	0.8 ± 0.2b	1.44 ± 0.15a	0.83 ± 0.18b
85	67.42	Phytol	150–86-7	2113	2114	MS,RI,STD	–	2.83 ± 0.52a	–

MS—identification based on the NIST 20 mass spectral database; ^a^ RI—retention index of each volatile on the TG-5MS column (30 m × 0.25 mm × 0.25 μm); ^b^ RI—retention index based on the NIST database. STD—means the compound was identified by standards. All data are expressed as mean ± S.D. (*n* = 5).

**Fig. 3:**
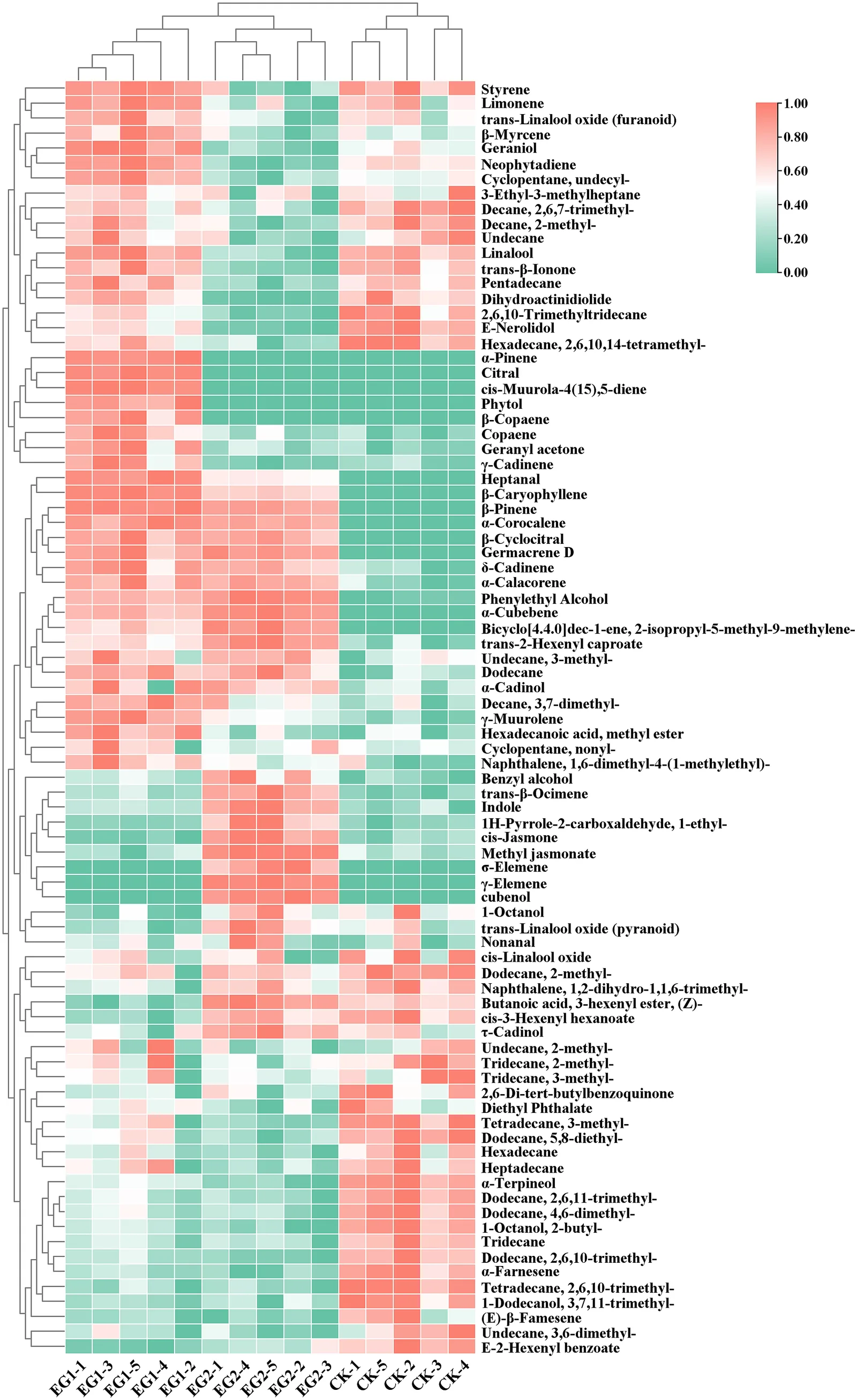
Heat map analysis of the concentrations of the main volatile compounds in CK, EG1 and EG2 green tea. (For interpretation of the references to colour in this figure legend, the reader is referred to the web version of this article.)

The 85 volatile compounds were classified into seven categories according to their functional groups, including 23 terpenes, 26 alkanes, 16 alcohols, 8 esters, 4 aldehydes, 4 ketones, and 4 other compounds (Fig. 4A). The distribution of volatile compounds in the three cultivation groups was illustrated in a Venn diagram (Fig. 4B). A total of 82, 80, and 69 volatile compounds were identified in EG1, EG2, and CK, respectively. Among them, 69 compounds were common to all three groups. Five and three compounds were exclusively detected in EG1 and EG2, respectively. In addition, eight compounds were shared by EG1 and EG2. EG1 exhibited the highest total volatile content, followed by EG2 and CK (Fig. 4C). These results indicate that intercropping treatments increased the diversity of volatile components. The corresponding ion chromatograms are presented in Fig. S3. The EI mass spectra of representative volatile compounds are presented in Fig. S4.

**Fig. 4:**
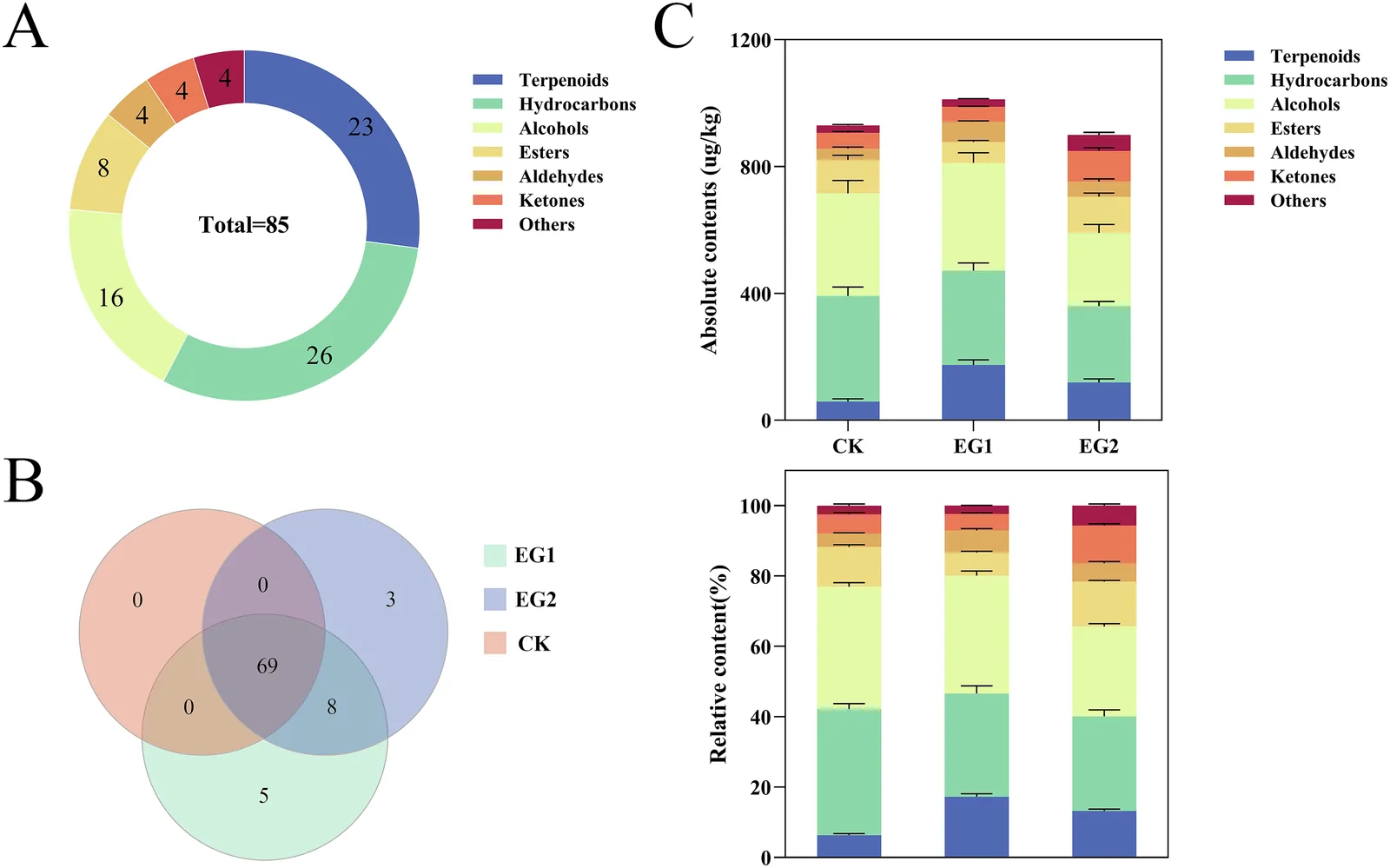
Volatile profiling of tea samples with different treatments. (A) Quantitative distribution of volatile compound categories (total = 85 compounds). (B) Venn diagram. (C) Stacked bar graphs for relative concentration (μg/kg) and relative proportion of seven volatile chemical classes.

Alcohols and hydrocarbons were the predominant volatile constituents in all three groups, accounting for more than 50% of the total volatile content (Fig. 4C). EG1 contained higher proportions of both alcohols and hydrocarbons than EG2. Terpenes accounted for more than 15% of the total volatiles in EG1, representing a higher proportion than that observed in EG2. In contrast, ketones and esters each accounted for more than 10% of the total volatile compounds in EG2, exceeding the corresponding proportions in EG1. Relative to CK, intercropping was associated with increased proportions of terpenes and aldehydes in EG1, whereas ketones were more enriched in EG2. These differences indicate that the two intercropping systems were associated with distinct volatile profiles. The higher abundance of terpenes and aldehydes in EG1 may have contributed to its floral, citrus, and green aroma characteristics, whereas the greater proportions of ketones and esters in EG2 may have contributed to its distinct aroma profile (Ma et al., 2025; Zhai et al., 2022). Although differences in canopy microenvironment and plant–plant interactions have been proposed as potential factors influencing volatile metabolism under intercropping conditions, environmental variables such as light intensity, temperature, and humidity were not directly measured in the present study. Therefore, the contribution of these ecological factors to the observed volatile differences cannot be established from the current data and should be considered a potential explanation rather than a demonstrated mechanism. Overall, the results demonstrate that intercropping was associated with shifts in the composition and relative abundance of tea volatiles, with EG1 and EG2 exhibiting distinct volatile characteristics.

Although the two companion species exhibited distinct volatile-associated metabolic profiles, the present data do not demonstrate that volatile emissions from the companion plants were transferred to or directly regulated flavor formation in tea. Likewise, the contribution of canopy structure, microclimate, and below-ground interactions cannot be individually resolved. Future studies using non-aromatic companion species with comparable canopy architecture, root-barrier treatments, and direct monitoring of volatile emissions and exchange would be valuable for disentangling these mechanisms.

### 3.5 Analysis of key volatile compounds

The volatile profiles of Lushan Yunwu tea under different cultivation systems were further evaluated by multivariate statistical analysis. The PCA score plot (Fig. 5A) explained 51% and 35.4% of the total variance along PC1 and PC2, respectively. The three treatment groups were clearly separated, indicating distinct differences in volatile composition among the cultivation systems. To further improve group discrimination, a supervised partial least-squares discriminant analysis (PLS-DA) was performed (Fig. 5B). The model showed clear separation among the sampled groups; however, given the limited independent biological replication, the PLS-DA results should be interpreted as exploratory rather than as evidence of a robust predictive model. Cross-validation was performed to verify model robustness (Fig. 5C). The resulting intercepts of R^2^ (0.0368) and Q^2^ (−0.41) confirmed the reliability of the PLS-DA model.

**Fig. 5:**
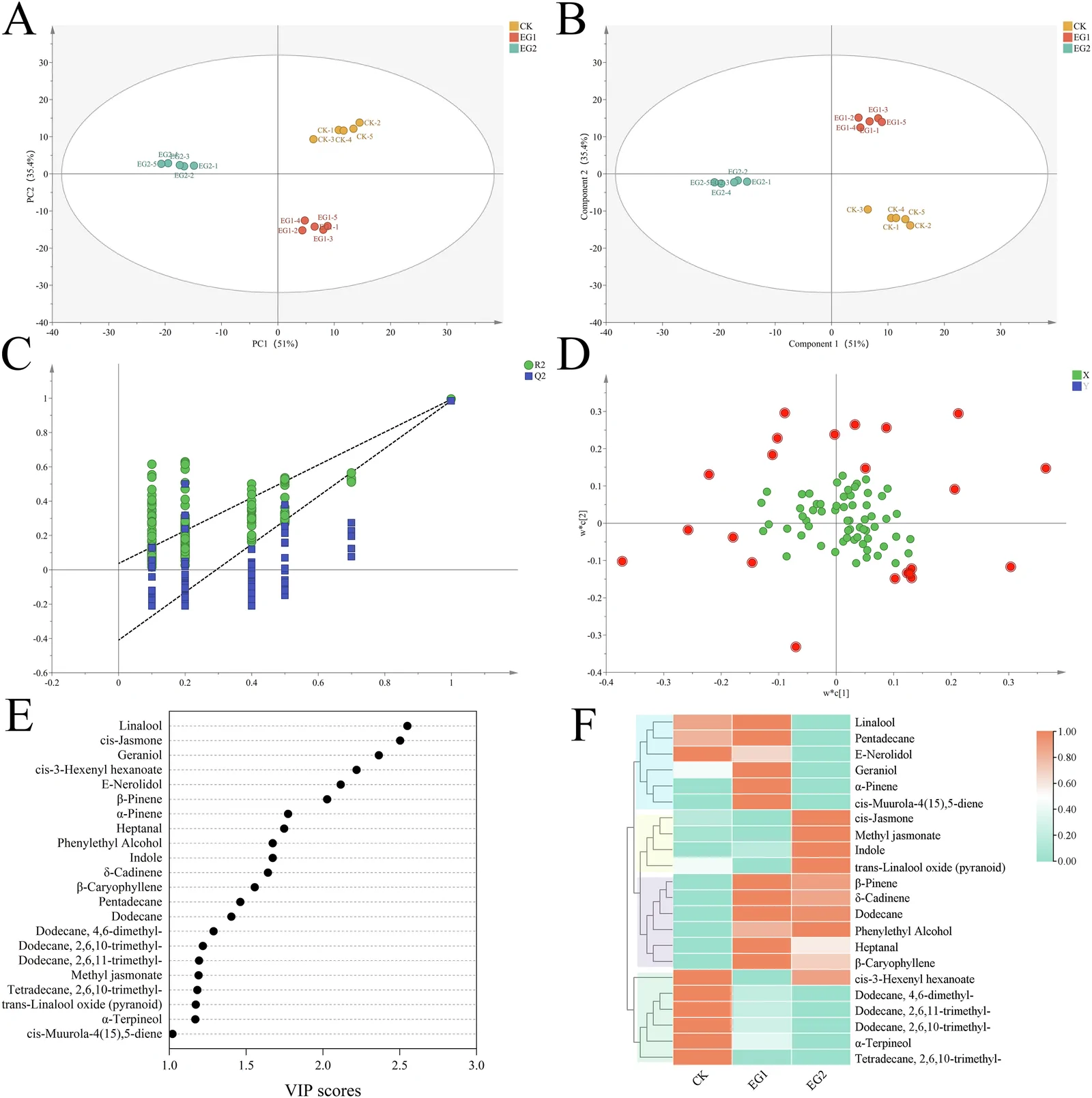
Multivariate analysis of volatile compound contents in CK, EG1 and EG2 green tea. (A) Principal component analysis scores, (B) partial least-squares discriminant analysis scores, (C) permutation test, (D) loading plots (red and green dots represent different compounds; red dots represent the most differential compounds, VIP >1), (E) VIP scores of volatile compounds (VIP > 1), (F) Heatmap of 22 differential volatile compounds. (For interpretation of the references to colour in this figure legend, the reader is referred to the web version of this article.)

The PLS-DA loading plot (Fig. 5D) further illustrates the contribution of individual volatile compounds to sample discrimination, with each point representing a single volatile compound. Variables located farther from the origin exerted greater influence on sample discrimination. Based on a variable importance in projection (VIP) value >1, a total of 22 differential volatile compounds were identified (Fig. 5E). Among them, linalool exhibited the highest VIP value (2.55) and reached its greatest concentration (101.19 μg/kg) in EG1. In addition, β-pinene (pine and turpentine aroma) and heptanal (fruity aroma) were detected only in the intercropping treatments (EG1 and EG2), while α-pinene (woody and herbal odor) and cis-muurola-4(15),5-diene were uniquely detected in EG1. These treatment-specific volatile compounds may contribute to the distinct aroma characteristics associated with different intercropping treatments.

To further visualize the distribution patterns of these differential volatile compounds, hierarchical clustering analysis was performed, and the resulting heatmap is shown in Fig. 5F. The 22 differential volatiles were broadly classified into four clusters according to their accumulation patterns across the three intercropping regimes. The first cluster (blue) comprised linalool, pentadecane, (*E*)-nerolidol, geraniol, α-pinene, and cis-muurola-4(15),5-diene, most of which accumulated predominantly in EG1. Among these, the terpene alcohols linalool, geraniol, and (E)-nerolidol are well-recognized contributors to floral aroma. The second cluster (yellow) included cis-jasmone, methyl jasmonate, indole, and trans-linalool oxide (pyranoid), which were highly enriched in EG2 and are associated with floral, sweet, jasmine-like, and woody aroma notes. The third cluster (purple) consisted of β-pinene, δ-cadinene, dodecane, phenylethyl alcohol, heptanal, and β-caryophyllene. These compounds were present at relatively high concentrations in both EG1 and EG2 and collectively contributed roasted, woody, floral, fruity, and spicy aroma characteristics. The fourth cluster (green), including cis-3-hexenyl hexanoate, 4,6-dimethyldodecane, 2,6,11-trimethyldodecane, 2,6,10-trimethyldodecane, α-terpineol, and 2,6,10-trimethyltetradecane, was predominantly enriched in CK. Overall, these clustering patterns indicated that *O. fragrans* intercropping system was characterized by higher abundances of several floral terpene-derived volatiles, whereas the monoculture system retained a volatile profile dominated by hydrocarbons, which generally contribute less to the characteristic aroma of green tea.

### 3.6 ROAVs analysis of key volatile compounds

The ROAVs of the major volatile compounds identified in CK, EG1, and EG2 are summarized in Table 3. Compounds with ROAVs >0.1 were considered to contribute substantially to the overall aroma, with higher values indicating greater sensory contributions. In CK, the characteristic aroma was primarily attributed to linalool (1.68), δ-cadinene (0.83), and geraniol (0.35), which collectively contributed to the typical fresh and herbal aroma of conventional green tea. Compared with CK, EG1 exhibited significantly higher ROAVs of linalool, δ-cadinene, and geraniol, together with the appearance of heptanal (ROAV = 0.48), which was not detected in CK.

**Table 3:** Relative odor activity values (ROAVs) of 12 volatile compounds with ROAV >0.1 in at least one sample.

Odorants	Odor Description	OT(μg/kg)	ROAV		
			CK	EG1	EG2
β-Myrcene	Woody, resinous, musty	1.2	0.06 ± 0.01b	0.11 ± 0.02a	0.08 ± 0.03b
Limonene	Citrus, lemon, orange-like, green	4	0.1 ± 0.01b	0.12 ± 0.01b	0.15 ± 0.02a
δ-Cadinene	Woody, herbal, terpene, spicy	1.5	0.83 ± 0.09c	1.77 ± 0.3b	2.95 ± 0.27a
Linalool	Floral, sweet, grape-like, woody	3.8	1.68 ± 0.04a	1.85 ± 0.16a	1.36 ± 0.19b
Geraniol	Rose-like, sweet, honey-like	7.5	0.35 ± 0.04b	0.62 ± 0.08a	0.39 ± 0.05b
cis-3-Hexenyl hexanoate	Fruity, waxy, green, fatty, winey	16	0.35 ± 0.01b	0.19 ± 0.02c	0.59 ± 0.07a
Methyl jasmonate	Floral, jasmine, green	3	0.09 ± 0.02b	0.08 ± 0.03b	0.67 ± 0.07a
Heptanal	Penetrating fruity odor	3	–	0.48 ± 0.1a	0.21 ± 0.02b
Nonanal	Floral, fatty, green, lemon-like	1.1	2.32 ± 0.22b	2.37 ± 0.21b	4.69 ± 0.89a
cis-Jasmone	Floral, woody, herbal, citrus, jasmine-like	7	0.39 ± 0.07b	0.32 ± 0.04b	1.62 ± 0.2a
trans-β-Ionone	Violet, raspberry, floral	0.007	100 ± 0	100 ± 0	100 ± 0
Naphthalene, 1,2-dihydro-1,1,6-trimethyl-	Licorice-like	2.5	0.24 ± 0.02b	0.17 ± 0.02c	0.39 ± 0.05a

OT—odor threshold in water based on the literature (Cui et al., 2022; Guo et al., 2022; Wang et al., 2025).

Heptanal is an important lipid-derived aldehyde generated through the lipoxygenase pathway during tea processing. Heptanal is widely recognized for imparting fresh, fruity, and green aroma notes (Wang et al., 2024). Previous studies have identified heptanal as a key odor-active compound in high-quality green tea, with its abundance closely associated with aroma freshness and richness (Zhang et al., 2024). The elevated ROAV of heptanal in EG1 (0.48) likely contributed to the enhanced freshness and greater aromatic complexity observed in this treatment.

In contrast, EG2 exhibited the highest ROAVs of cis-jasmone (1.62) and methyl jasmonate (0.67) among the three groups. *Cis*-Jasmone is commonly identified in green tea, imparting a jasmine-like aroma even at low concentrations (Liu et al., 2025; Zhu et al., 2016). Methyl jasmonate, a key terpenoid ester, contributes elegant orchid-like floral notes and enhances aroma complexity in premium teas (Guo et al., 2022; Zhu et al., 2015). The high ROAVs of these two compounds indicate that jasmonate-related volatiles were major contributors to the distinctive floral aroma of EG2. Notably, δ-cadinene also reached its maximum ROAV (2.95) in EG2, enriching the woody and herbal terpene characteristics of tea infusion. In summary, *O. fragrans* intercropping primarily enhanced the characteristic aroma of green tea, whereas *P. serratifolia* intercropping developed distinct floral and woody notes.

This study constructed a multi-parametric characteristic profile of Lushan Yunwu tea based on the quality-related physicochemical, taste, and aroma parameters investigated, and identified 19 taste-active compounds and eight aroma-active compounds that were closely associated with the observed quality differences among the tea samples. (Fig. 6). Taken together, the sensory differences among CK, EG1, and EG2 were associated with coordinated changes in both non-volatile taste compounds and volatile aroma compounds. EG1 formed a balanced fresh-floral profile, whereas EG2 exhibited a more pronounced bitter-astringent and jasmine-like profile. From an agronomic perspective, the two aromatic woody species demonstrated distinct application potential. *O. fragrans* intercropping appears more suitable for producing premium green tea with enhanced freshness and floral aroma, whereas *P. serratifolia* intercropping may be more appropriate for developing teas with intense floral characteristics. One limitation of the present study is that, although fresh shoots were collected for green-tea processing, matched fresh-leaf samples were not preserved for parallel chemical and sensory-related analyses. Therefore, the present dataset does not allow direct comparison between metabolite profiles before and after processing or evaluation of whether the observed differences were already present in fresh leaves. Future studies should preserve matched fresh-leaf samples from each cultivation system for parallel metabolomic and volatile analyses.

**Fig. 6:**
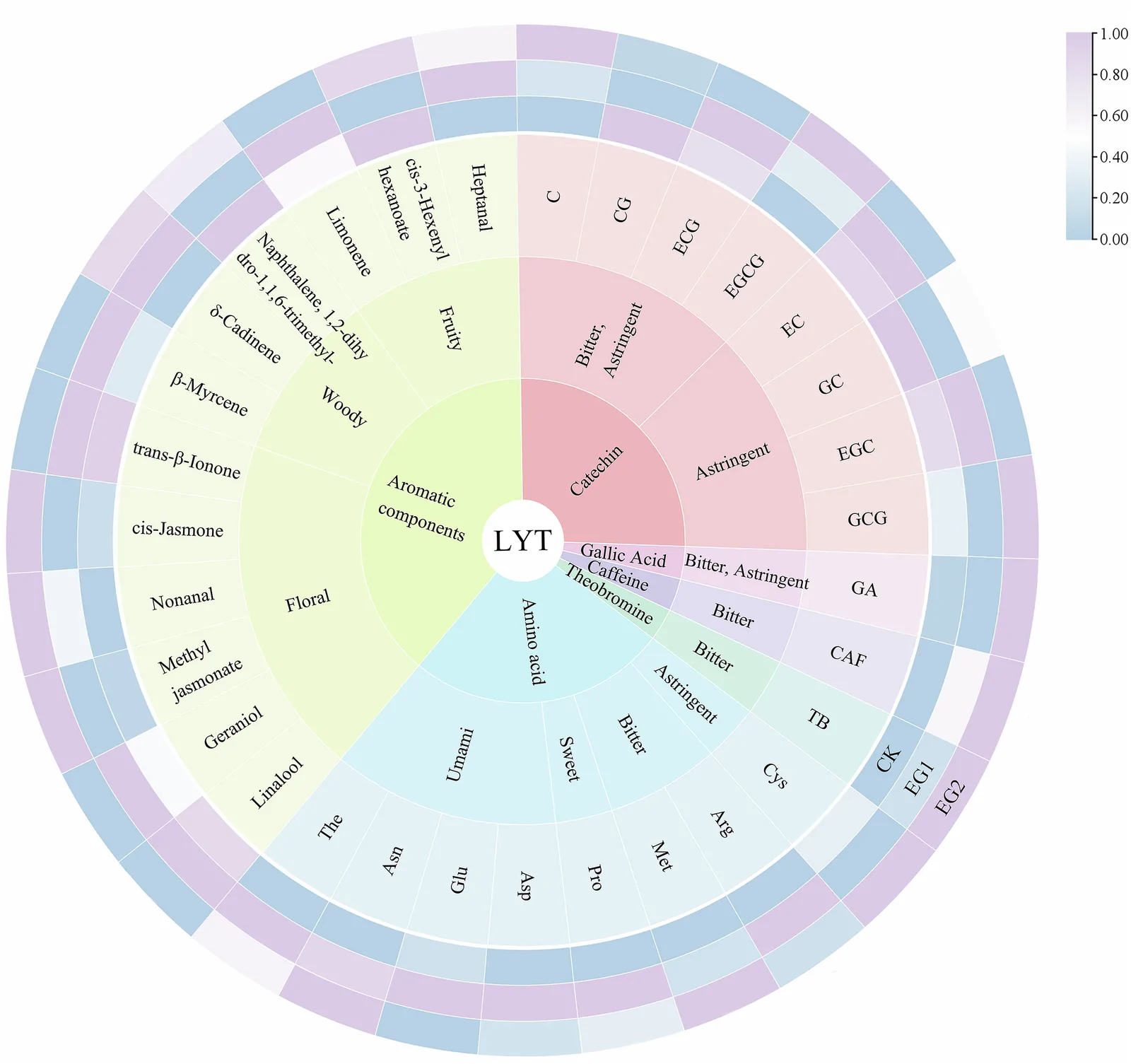
Flavor wheel of key substances with TAV > 1 and ROAV >0.1. Note: The heatmap represents the relative content of substances. C, catechin; CG, catechin gallate; ECG, epicatechin gallate; EGCG, epigallocatechin gallate; EC, epicatechin; GC, gallocatechin; EGC, epigallocatechin; GCG, gallocatechin gallate; GA, gallic acid; CAF, caffeine; TB, theobromine; The, theanine; Asn, asparagine; Glu, glutamate; Asp, aspartate; Pro, proline; Met, methionine; Arg, arginine; Cys, cysteine.

## 4 Conclusion

This study investigated the effects of two aromatic woody intercropping systems on the flavor quality of green tea by integrating sensory evaluation with analyses of taste-related metabolites and volatile compounds. Both intercropping systems significantly reshaped the flavor profile of green tea but exhibited distinct regulatory patterns. *O. fragrans* intercropping enhanced the concentrations of umami- and sweet-tasting amino acids, non-esterified catechins, and floral terpene alcohols, thereby contributing to improved freshness, floral aroma, and overall sensory quality. In contrast, *P. serratifolia* intercropping preferentially increased the levels of esterified catechins, caffeine, gallic acid, and jasmonate-related volatile compounds, leading to stronger bitterness and astringency together with a distinctive jasmine-like aroma profile. Integrated analyses of TAVs and ROAVs further demonstrated that the flavor differences among the three cultivation systems were associated with coordinated changes in both taste-active metabolites and aroma-active volatiles. These findings indicate that different companion-tree systems were associated with distinct tea flavor profiles and metabolite patterns. Under the conditions examined in this study, *O. fragrans* intercropping was associated with a fresher and more floral sensory profile, whereas *P. serratifolia* intercropping was associated with stronger bitter-astringent and floral characteristics. Overall, this study provides new insights into how different companion tree species influence tea flavor formation under intercropping systems and offers a theoretical basis for companion species selection and precision management in sustainable tea agroforestry systems.

## CRediT authorship contribution statement

**Yanan Liu:** Writing – original draft. **Yanlu He:** Writing – original draft. **Liping Yao:** Data curation. **Xiangqun Shi:** Data curation. **Jiaxin Liu:** Investigation. **Li Zhang:** Investigation. **Guizhen Chen:** Data curation. **Changshuai Yu:** Investigation. **Jigang Huang:** Investigation. **Yinxiang Gao:** Writing – review & editing, Conceptualization.

## Informed consent statement

Ethical approval for the sensory evaluation involving five participants was obtained from the Ethics Committee of Jiujiang University. And all sensory evaluators agreed to participate and signed the in- formed consent form.

## Institutional review board statement

Sensory evaluation data collection and use were in accordance with Jiujiang University Human Ethics application ID Number: JJUM20240090 (Approval Date: 19 April 2024).

## Declaration of competing interest

The authors declare that they have no known competing financial interests or personal relationships that could have appeared to influence the work reported in this paper.

## Data Availability

Data will be made available on request.
