# Supplementary material for: Species-dependent variation in flavor profile and sensory quality of green tea under aromatic woody intercropping systems
Source: Food Chem X. 2026 Oct 16;40:104647. doi: 10.1016/j.fochx.2026.104647 (PMC13695123; doi:10.1016/j.fochx.2026.104647)
Supplement: Supplementary file 1 [file mmc1.docx]

**Chemicals**

CAF (≥98%), gallic acid (GA, ≥99%), theobromine (TB, ≥98%), and catechin standards, including epigallocatechin gallate (EGCG), epicatechin gallate (ECG), epigallocatechin (EGC), gallocatechin (GC), catechin (C), epicatechin (EC), gallocatechin gallate (GCG), and catechin gallate (CG) (all ≥98%) were purchased from Solarbio Technology Co., Ltd. (Beijing, China). Similarly, amino acid standards including L-serine (Ser), L-histidine (His), L-methionine (Met), L-aspartic acid (Asp), L-alanine (Ala), γ-aminobutyric acid, L-arginine (Arg), L-glutamic acid (Glu), L-glycine (Gly), L-isoleucine (Ile), L-leucine (Leu), L-lysine (Lys), L-phenylalanine (Phe), L-proline (Pro), L-threonine (Thr), L-tyrosine (Tyr), L-valine (Val), and L-theanine (The) (all HPLC grade, ≥98%), were also purchased from Solarbio Technology Co., Ltd. (Beijing, China). HPLC-grade acetonitrile and methanol were purchased from Merck (Darmstadt, Germany). Analytical reagents including acetic acid (99.8%), disodium EDTA (≥99%, chromatography grade), ascorbic acid (≥99.99%), and triethylamine (≥99.5%, chromatography grade), and volatile compound standards, including cis-3-hexenyl hexanoate (98%), hexadecanoic acid methyl ester (99%), benzyl alcohol (≥99.5%), linalool (98%), phenylethyl alcohol (>99.5%), α-terpineol (>95.0%), geraniol (≥99.0%), (E)-nerolidol (≥95%), phytol (>90.0%), nonanal (97%), cis-jasmone (98%), geranyl acetone (97%), β-myrcene (≥90.0%), β-caryophyllene (≥98%), and α-farnesene (98%), were supplied by Aladdin Biochemical Technology Co., Ltd. (Shanghai, China). Ethyl decanoate (99%) was used as the internal standard for volatile compound analysis and were supplied by Aladdin Biochemical Technology Co., Ltd. (Shanghai, China). A C7–C40 n-alkane mixture was obtained from Sigma-Aldrich (Shanghai, China). Ultrapure water was produced using a Milli-Q purification system (Millipore, Bedford, MA, USA). An AccQ-Tag derivatization kit for amino acid analysis (WAT052880) was purchased from the Waters Corporation (Milford, MA, USA).

**Supplementary Method 1: Extraction and analysis of amino acids using UPLC**

A 40 mg sample of tea powder was measured, and 8 mL of ultrapure water was added, followed by 10s of vortexing. The mixture underwent ultrasonic treatment in a water bath at room temperature for 30 min, then was centrifuged at 4°C at 10,000 rpm for 5 min. The resulting supernatant was filtered through a 0.22 µm microporous membrane. For amino acid separation, the FLD-3x00 (RS) fluorescence detector replaced the DAD-3000 (RS) diode array detector described in section 2.4.1, and a C18 column 100 × 2.1 mm, 1.7 µm (Syncronis, Thermo Fisher, USA) was utilized. The remaining modules and operational settings of the HPLC system were consistent with those described in Section 2.4. The column was maintained at 35 °C, and the fluorescence detector was set to 45°C. Detection was performed using an excitation wavelength of 250 nm and an emission wavelength of 395 nm, with the Xenon lamp in LongLife mode and sensitivity set to three. The flow rate was 0.4 mL/min, with an injection volume of 2 μL. Mobile phase A was prepared by combining 95 mL of potassium dihydrogen phosphate, 5 mL of disodium hydrogen phosphate, and 10 mL of triethylamine hydrochloride solution, with water added to reach a total volume of 1 L. The mobile phase B consisted of 100 mL of potassium dihydrogen phosphate, 250 mL of water, and 500 mL of acetonitrile, which was then added with water to make up the volume of 1L. The gradient elution conditions are as follows: 0-1.70 min, B: 0%; 1.70-8.50 min, B: 0%-2.7%; 8.50-13.60 min, B: 2.7%-18.8%; 13.60-18.70 min, B: 18.8%-25.6%; 18.70-22.78 min, B: 25.6%-49.8%; 22.78-23.80 min, B: 49.8%-100%; 23.80-25.50 min, B: 100%; 25.50-26.18 min, B: 100%-0%. The individual amino acid was verified by comparing their retention time with that of standard substances and quantified based on the regression equation of the corresponding calibration curve.

**Supplementary Method 2:** **Extraction and analysis of catechins, theobromine, and caffeine using HPLC**

A 0.2 g sample of tea powder was placed in a 10 mL centrifuge tube, followed by the addition of 5 mL of 70% (v/v) methanol heated to 70°C. The mixture was vortexed for 10 s, and then subjected to oscillation extraction in a water bath at 70°C for 10 min. Afterward, the sample was vortexed again for 10 s and treated ultrasonically in a water bath for 5 min. The mixture was centrifuged at 10,000 rpm for 5 min, and the resulting supernatant was transferred to a 10 mL volumetric flask. Before analysis, the solution was filtered through a 0.22 µm membrane. The method was adapted and optimized from a previously reported procedure (Dai et al., 2018; Ma et al., 2022). The HPLC system used comprised a DGP-3600RS dual ternary pump, WPS-3000 auto-sampler, TCC-3x00 (RS) column oven, and a DAD-3000 (RS) diode array detector (Ultimate 3000, Thermo Fisher, USA). Separation of individual catechins, caffeine, and theobromine was performed using a Phenyl column 250 × 4.6 mm, 5 µm (Hypersil GOLD, Thermo Fisher, USA). Mobile phase A consisted of 9% (v/v) acetonitrile, 2% (v/v) acetic acid, and 20 µg/mL EDTA-2Na, while mobile phase B was composed of 80% acetonitrile, 2% acetic acid, and 20 µg/mL EDTA-2Na. The gradient elution program was as follows: 0–10 min, B: 0%; 10–24 min, B: 0%–32%; 24–26 min, B: 32%; 26–27 min, B: 32%–0%. The flow rate was set to 1 mL/min, with the column maintained at 35 °C and an injection volume of 10 µL. Absorbance at 278 nm was recorded to monitor peak intensity, with peak identification achieved by comparing retention times to those of standards. Quantification was based on calibration curve regression.

**Supplementary Method 3: Extraction and analysis of volatile compounds using HS-SPME-GC-MS**

1. *Extraction of Volatile Compounds from Tea Using HS-SPME*

Volatile compounds were extracted using HS-SPME with a divinylbenzene/carboxen/polydimethylsiloxane coating fiber (50/30 μm; 1 cm) purchased from Supelco (Bellefonte, PA, USA). All tea samples were ground, passed through 40 mesh, and sealed for future use. Briefly, ground tea powder (1.5 g) was accurately weighed into a 20 mL headspace flask. Then, NaCl (0.5 g) was added, followed by 20 μL of ethyl decanoate (20 μg/mL) as an internal standard, and 5.25 mL of distilled water. Subsequently, the autosampler was stabilized to ensure the injection stability. The flask was incubated at 70°C for 30 min with shaking (5 seconds on, 2 seconds off). The coating fiber was then inserted into the headspace of the flask for extraction at 70°C for 30 min. Finally, the fiber was inserted into the injector port of the gas chromatography-mass spectrometry (GC-MS) for desorption at 250°C for 5 min, after which GC-MS separation and identification were performed.

*2. Gas Chromatography-Mass Spectrometry Analysis*

GC-MS was performed using a Thermo TSQ 8000 spectrometer equipped with a TriPlus RSH three-in-one automatic sampler TPR (Thermo Fisher Scientific, Waltham, MA, USA). TG-5MS gas chromatography column with 30 m×0.25 mm×0.25 μm film thickness (Thermo Fisher, USA) was used for separation and high-purity helium (≥99.999%) was used as carrier gas. The constant flow rate was 1.0 mL/min, and the injector temperature was 280°C. The mass spectrometry conditions were as follows: electron bombardment ion source (EI), ion source temperature of 300°C, mass interface temperature of 280°C, and ionization voltage of 70 eV. The mass scanning range was 33 to 550 m/z. The initial oven temperature was maintained at 40°C for 2 min and then increased at 5°C /min to 85°C, where it was held for 2 min. Subsequently, the temperature was increased at a rate of 2°C/min to 110°C and held for 2 min, then increased at a rate of 4°C/min to 220°C, where it was held for 2 min, and finally increased at 5°C/min to 250°C and held for 10 min.
