# Supplementary material for: Species-dependent variation in flavor profile and sensory quality of green tea under aromatic woody intercropping systems
Source: Food Chem X. 2026 Oct 16;40:104647. doi: 10.1016/j.fochx.2026.104647 (PMC13695123; doi:10.1016/j.fochx.2026.104647)
Supplement: Supplementary file 2 [file mmc2.docx]

Table S1 Description of sensory evaluation in CK, EG1 and EG2 green tea samples.

| **Samples** | **Appearance of dry tea leaf**  **(25%)** | | **Liquor Color**  **(10%)** | | **Aroma of tea infusion**  **(25%)** | | **Taste**  **(30%)** | | **Appearance of infused leaves (10%)** | | **Total Score** |
| --- | --- | --- | --- | --- | --- | --- | --- | --- | --- | --- | --- |
|  | **Evaluation** | **Score** | **Evaluation** | **Score** | **Evaluation** | **Score** | **Evaluation** | **Score** | **Evaluation** | **Score** |  |
| CK | Bright yellow and green, tight and thin | 91±1 | Yellowish green, bright | 90.1±0.55 | Fresh and clear | 90±0.71 | Fresh and heavy | 90±0.71 | Yellowish green, bright | 90.4±0.55 | 90.3±0.41 |
| EG1 | Bright yellow and green, tight and thin | 91.2±0.84 | Yellowish green, bright | 90.2±0.84 | Fresh and clear, mild flowery  aroma | 90.2±0.84 | Fresh and heavy, returning sweetness | 92±0.71 | Yellowish green, bright | 91.4±0.89 | 91.11±0.48 |
| EG2 | Bright yellow and green, tight and thin | 90.8±0.84 | Yellowish green, bright | 90.2±0.84 | Fresh, flowery  aroma obvious | 91.6±0.55 | Fresh and heavy | 89.8±0.84 | Yellowish green, bright | 90.6±0.55 | 90.62±0.29 |

Table S2 The content of amino acids in CK, EG1 and EG2 green tea samples (mg/g).

|  | Umami amino acids | Sweet-tasting amino acids | Bitter-tasting amino acids | Total amino acids |
| --- | --- | --- | --- | --- |
| CK | 14.71±0.13 | 3.25±0.22 | 5.1±0.13 | 24.78±0.65 |
| EG1 | 21.18±0.72 | 5±0.27 | 6.94±0.2 | 33.49±1.08 |
| EG2 | 17.24±1.22 | 3.37±0.15 | 8.57±0.43 | 29.8±1.84 |

Table S3 The content of catechin in CK, EG1 and EG2 green tea samples (mg/g).

|  | Non-esterified catechins | Esterified catechins | Total catechins | Esterified catechins/  Non-esterified catechins |
| --- | --- | --- | --- | --- |
| CK | 14.1±1.35 | 41.65±5.013 | 55.75±6.38 | 2.95±0.07 |
| EG1 | 18.06±0.32 | 47.6±1.17 | 65.66±1.48 | 2.63±0.02 |
| EG2 | 5.49±0.13 | 61.23±2.22 | 66.73±2.34 | 11.15±0.19 |

Table S4 The absolute contents and percentages relative to total amino acids of Glu, Asp, The and Arg in the CK, EG1, and EG2 groups.

| Groups | Compound | Absolute contents (mg/g) | Percentage（%） |
| --- | --- | --- | --- |
| CK | Glu | 2.91±0.04 | 11.76% |
|  | Asp | 1.59±0.02 | 6.41% |
|  | The | 9.12±0.02 | 36.80% |
|  | Pro | 0.51±0.19 | 2.07% |
| EG1 | Glu | 4.11±0.13 | 12.26% |
|  | Asp | 2.21±0.04 | 6.61% |
|  | The | 13.7±0.68 | 40.88% |
|  | Pro | 1.34±0.08 | 4.01% |
| EG2 | Glu | 2.7±0.15 | 9.08% |
|  | Asp | 1.7±0.1 | 5.72% |
|  | The | 11.66±0.99 | 39.10% |
|  | Pro | 0.75±0.03 | 2.51% |
